# Cutting-Edge Technologies for Inflamed Joints on Chip: How Close Are We?

**DOI:** 10.3389/fimmu.2022.802440

**Published:** 2022-03-10

**Authors:** Emine Kahraman, Ricardo Ribeiro, Meriem Lamghari, Estrela Neto

**Affiliations:** ^1^ Instituto de Engenharia Biomédica (INEB), Universidade do Porto, Porto, Portugal; ^2^ Instituto de Investigação e Inovação em Saúde (i3S), Universidade do Porto, Porto, Portugal; ^3^ Faculdade de Engenharia da Universidade do Porto (FEUP), Rua Dr. Roberto Frias, Porto, Portugal

**Keywords:** microfluidic, joint-on-a-chip, osteoarthritis, 3D bioprinting, Cartilage, tissue engineering

## Abstract

Osteoarthritis (OA) is a painful and disabling musculoskeletal disorder, with a large impact on the global population, resulting in several limitations on daily activities. In OA, inflammation is frequent and mainly controlled through inflammatory cytokines released by immune cells. These outbalanced inflammatory cytokines cause cartilage extracellular matrix (ECM) degradation and possible growth of neuronal fibers into subchondral bone triggering pain. Even though pain is the major symptom of musculoskeletal diseases, there are still no effective treatments to counteract it and the mechanisms behind these pathologies are not fully understood. Thus, there is an urgent need to establish reliable models for assessing the molecular mechanisms and consequently new therapeutic targets. Models have been established to support this research field by providing reliable tools to replicate the joint tissue *in vitro*. Studies firstly started with simple 2D culture setups, followed by 3D culture focusing mainly on cell-cell interactions to mimic healthy and inflamed cartilage. Cellular approaches were improved by scaffold-based strategies to enhance cell-matrix interactions as well as contribute to developing mechanically more stable *in vitro* models. The progression of the cartilage tissue engineering would then profit from the integration of 3D bioprinting technologies as these provide 3D constructs with versatile structural arrangements of the 3D constructs. The upgrade of the available tools with dynamic conditions was then achieved using bioreactors and fluid systems. Finally, the organ-on-a-chip encloses all the state of the art on cartilage tissue engineering by incorporation of different microenvironments, cells and stimuli and pave the way to potentially simulate crucial biological, chemical, and mechanical features of arthritic joint. In this review, we describe the several available tools ranging from simple cartilage pellets to complex organ-on-a-chip platforms, including 3D tissue-engineered constructs and bioprinting tools. Moreover, we provide a fruitful discussion on the possible upgrades to enhance the *in vitro* systems making them more robust regarding the physiological and pathological modeling of the joint tissue/OA.

## 1 Introduction

Osteoarthritis (OA) is the most common joint arthritis and it is caused by the breakdown of connective tissue. According to World Health Organization (WHO), 9.6% of men and 18.0% of women aged over 60 years are diagnosed with OA and the majority of these patients have limitations in their daily activities (1). OA eventually leads to a complete destruction of the articular layer and surgery is the only recommended treatment to improve patient’s quality of life (2). Despite the great impact of the disease, the large number of patients and some known associated risk factors (age, gender, obesity, genetics) (1), there is still no effective treatment for OA and cartilage repair.

Joint tissue is a complex structure that is comprised of articular cartilage, subchondral bone, synovium and synovial fluid, ligaments and menisci (3). Articular cartilage is an avascular and non-innervated connective tissue, that covers the end of long bones to provide frictionless movement and includes high amount of extracellular matrix (ECM) (4). Other components of the joint, such as synovium and synovial fluid, are critical for nutrition supply to the joint system (5). Synovium consists of synoviocytes [also called fibroblast-like synoviocytes (FLS)] which are responsible for secreting lubricin, meniscus cells and macrophages. This tissue plays an important role in balancing the joint homeostasis, both under inflammatory and healthy conditions (3, 5). For this reason, synovial inflammation is accepted as the main reason for musculoskeletal diseases, such as rheumatoid arthritis (RA) and OA (3). One of the main reasons is that there are many players taking a role in inflammatory joint pathologies. Dissecting the mechanisms behind this crosstalk is highly challenging and therefore, there is an urgent need to establish reliable models for assessing the molecular mechanisms and consequently new therapeutic targets.

Different *in vitro* models have been developed to investigate musculoskeletal diseases in the last few years. However, most of the *in vitro* models do not contemplate pain that occurs through the crosstalk between inflamed joint tissue and peripheral nervous system. In this review, we will shed light on the *in vitro* tools available to address the joint tissue in physiological and pathological conditions. We will provide the biological background pinpointing the crucial aspects that should be addressed when establishing appropriate cartilage tissue engineering-based *in vitro* models. We will follow through the available technological approaches used to establish *in vitro* cartilage constructs. We also aim to provide a discussion on healthy and inflamed joint tissue environment, OA associated pain, molecular players in the pain and currently developed *in vitro* models. This review addresses the pros and cons of currently available *in vitro* models to contribute to the development of innovative therapeutic targets that might improve the life quality of OA patients.

## 2 Biological Insight: Understanding Joint and Arthritis Environment

In healthy joint, articular cartilage is comprised of chondrocytes, the main cell type of cartilage tissue, which are located in a gel-like ECM, that is initially produced by chondroblasts. The ECM is mainly composed of collagen fibers, glycosaminoglycans (GAGs), aggrecan proteoglycan, and elastin fibers (6). To enhance cell-matrix interactions, chondrocytes are lied into highly organized collagen fibers. The orientation of those components and the interstitial fluid flow of cartilage affect the mechanical behavior of the tissue through pressure differences. In OA, under the compression, negatively charged aggrecan proteoglycans surrounded by sulfated GAGs, are pushed to each other generating a mutual repulsion force (7). These transitory changes in aggrecan charges are regulated by release of matrix-metalloproteinases (MMPs) and aggrecanases. This mechanism causes the breakdown of aggrecan proteins into proteoglycan macromolecules which results into loss of sulfated GAGs from the connective tissue (8).

Studies showed that multiple signaling pathways are involved both in healthy chondrocyte differentiation and OA by activation of hypertrophic differentiation of chondrocytes, such as WNT, Bone morphogenetic protein (BMP)/transforming growth factor-beta (TGFβ), Indian hedgehog (IHH), Fibroblast growth factor (FGF), Insulin like growth factor (IGF) and hypoxia-inducible factor (HIF) (9). Homeostasis between these pathways regulates chondrocytes phenotype in 3D, either they maintain chondrogenic phenotype or undergo hypertrophic differentiation that leads endochondral ossification or potentially OA development. Among the others, chondrocyte differentiation is mainly regulated by TGFβ family, which are subdivided in three: TGFβ1 that plays a key role on cell expansion, TGFβ2, while TGFβ3 is critical for redifferentiation of chondrocytes (10). The main intracellular signaling routes of this family is through the receptor-Smads that blocks the hypertrophy and terminal differentiation of chondrocytes (11). In early stage of chondrogenic differentiation, presence of TGFβ in culture medium, induce N-cadherin expression, MAP kinase activity and modulation of Wnt signaling, as well as promoting expression of ECM proteins (Col-II, SOX9 and ACAN) (11). In contrast to this, chondrocyte differentiation is inhibited upregulating the transcription factor Runx2.

Another TGFβ superfamily signaling protein is BMPs. BMPs are associated to almost all skeleton development stages and several are crucial during chondrogenic differentiation, such as BMP2, BMP3, BMP4, and BMP7. This chondrocyte differentiation is regulated by BMP-Smad pathway activation when specific BMPs bind to their receptors, followed by transferring the protein-receptor complex to the nucleus of chondrocytes to control the expression of SOX9 and Runx2 genes (12). More specifically, recent studies showed that BMP pathways frequently correlated with mixing numerous receptors and ligands (10). For example, combination of BMP9 and BMP10 on joint models, upregulates vascularization, while combination of combination BMP7 and TGFβ3 boost chondrogenic differentiation as each individual promotes the expression of cartilage ECM proteins (10). BMPs can also regulates proliferation of chondrocyte by up-regulating the expression of the Wnt and Notch signaling pathways.

Wnt signaling pathway is a highly essential pathway for chondrocyte proliferation and cartilage homeostasis. Wnt signaling pathway comprises of three well known intracellular signaling cascades: mitogen-activated protein kinase (MAPK), WNT/Ca2+ pathway and c-Jun N-terminal kinase (JNK) (9, 12). When Wnts bind to Wnt receptor, they form a complex that leads to release of β-catenin from the complex. However, excessive activation of Wnt pathway cause accumulation of the β-catenin that results with premature chondrocyte hypertrophy and expression of cartilage degrading metalloproteinases, Col-X and RUNX2, in other words cause to OA-like phenotype, while abolishing β-catenin from the articular cartilage promote expression of SOX9 and ACAN matrix proteins (9). Hence, Wnt signaling pathway has a key role as a regulator for chondrocytes phenotype during cartilage development depending on the which ligands included to the process. For example, Wnt3a, Wnt5a, Wnt5b promoting chondrogenic differentiation, while Wnt4, Wnt8 block chondrogenic differentiation and promote hypertrophy, and Wnt9a blocks both chondrogenic differentiation and hypertrophy (9). Another well-known ligand is that Wnt16 is also defined as promoter of hypertrophy, upregulation of Wnt16 is correlated with downregulation of Frizzled-related protein (FRZB), Gremlin 1 (GREM1) and Dickkopf-1 homolog (DKK1). Nevertheless, this is a complex process and during chondrogenic differentiation in 3D, multiple signaling pathways are involved simultaneously. Thus, as it is defined by Hu et al., many research groups have been working on the development of a growth factor mixture that can modulate the redifferentiation of chondrocytes.

**Figure 1:**
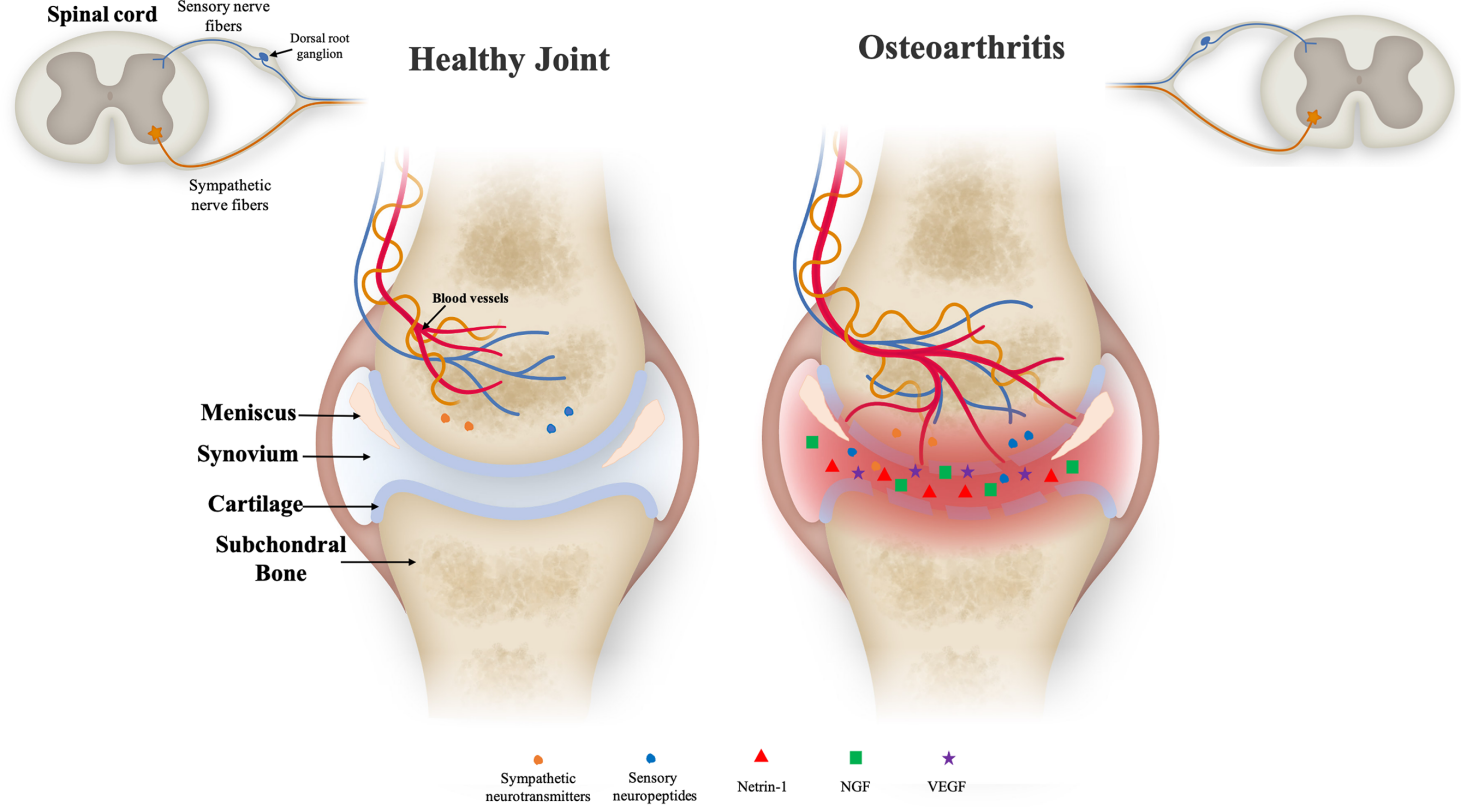
Illustration of the joint microenvironment on both healthy (left) and inflamed (right) conditions. Healthy cartilage is composed of well-defined layers of distinct zones as articular bone, cartilage layer, meniscus, synovial membrane and synovium. Nerve fibers and blood vessels are present in the periosteum, cortical bone and bone marrow, in relatively low density. In degenerative cartilage, the zones comprising the joint tissue are degraded and neuronal fibers are present showing higher density. The increased amount of blood vessels, sensory and sympathetic nerve fibers is into cartilage zone might be due to the pro-inflammatory environment and upregulation of neurotrophic factors as Netrin-1 and nerve growth factor (NGF) expression. These sensory nerve fibers are responsible for conducting the noxious stimuli to the spinal cord to be further processed by the central nervous system leading to the pain processing.

In OA, inflammation is frequent and controlled by the activation of upregulated Toll-like receptors (TLRs), innate immune cells (macrophages) and adaptive immune cells (T cells and B cells). These cells secrete pro-inflammatory cytokines such as Tumor necrosis factor-α (TNF-α), interleukin-1β (IL-1β) and IL-6 into the synovial membrane (13). These inflammatory cytokines then migrate into the cartilage and increase the release of the MMPs (MMP-1, MMP-3, MMP-13 and ADAMTS-5) and aggrecanases. This event leads to cartilage ECM degradation that includes mostly loss of aggrecan and collagen-II (Col-II) from the superficial zone, chondrocyte apoptosis and, finally necrosis (14, 15). Additionally, in the late stage of OA during inflammation, small pores appear at the chondral junction and allow the growth of neuronal fibers into subchondral bone (**Figure 1**). Later, sensory neuronal fibers contact with calcified cartilage zone that leads to pain, one of the most common clinical symptoms of OA (1, 16, 17). Even though pain in OA patients is mostly caused by upregulation or downregulation of above-mentioned cytokines in surrounding tissue microenvironment, it is described that chondrocytes at the superficial zone of articular cartilage produce nerve growth factor (NGF), vascular endothelial growth factor (VEGF) and platelet-derived growth factor (PDGF) under inflammatory conditions (16). The upregulation of NGF and VEGF by chondrocytes and other mediators from synovium, such as chemokine ligands (CCL) family and Netrin-1, an osteoclast-secreted protein, promote new nerve growth and vascularization (18). It has been reported that the level of vascularization in cartilage is correlated with the degree of innervation that leads to pain (19).

Although the above-mentioned data describe the role of inflammatory mediators in OA, additional research is necessary for enlightening the mechanism behind inflammation and to offer fully joint repair in long term period for the patients. To this regard, researchers developed disease modeling tools and drug testing devices using tissue engineering (TE) approaches (20). Hence, tissue engineering strategies pave the way to provide more reliable *in vitro* tools with the ultimate goal of developing new treatments for damaged joint tissue by combining both cellular and acellular approaches.

## 3 Tissue Engineering Approaches

### 3.1 Cell-Based Technologies

Chondrocytes *in vitro* cell culture were firstly performed in 2D systems (21), where monolayer cultures have been accepted as useful tools for studying cartilage biology in both normal and anomalous settings. Huang et al. reported that a 2D culture of articular chondrocytes that were subjected to mechanical stress to mimic OA displayed catabolic reactions such as increased expression of MMPs (22). Another study showed that monolayer cultures can also be used to study cartilage–synovium relation through exposing FLS to conditioned medium of chondrocytes (stimulated with leptin) in order to mimic the biochemical environment of OA on obese patients (23). However, it has been indicated that 2D monolayer cell culture systems do not fully recapitulate the biological stimulations since chondrocytes tend to dedifferentiate and lose their chondrogenic phenotype into fibroblastic-like cells (24). It is also indicated that chondrocytes in 2D have higher activity of Wnt/β-catenin signaling that results with accumulation of β-catenin (25). This leads to decreased synthesis of Col-II, aggrecan and proteoglycan to form cartilage ECM (26, 27). With respect to this, several studies have started to focus on the development of different culture strategies, including suspension cultures and coated surfaces (28). Nevertheless, these models still poorly mimic the complex joint environment. Hence, in the last decades, research studies have followed a path to recapitulate joint tissue microenvironment in 3D systems, both in healthy and inflamed conditions.

#### 3.1.1 Healthy Joint Model

Currently 3D cell-based strategies include micromass systems, also referred as 3D pellet formation. 3D pellet culture systems are obtained through suspension of different cell densites in a solution (culture medium or a buffer) and followed by centrifugation to form a material-free 3D cell aggregates in order to mimic cell-cell interactions in high cell density cell agglomerates (29, 30). Watts et al. studied cell survival, growth and chondrogenic capacity of mesenchymal stem cells (MSCs) comparing 3D pellet systems and agarose, alginate, and fibrin-alginate hydrogels (30). They demonstrated that MSCs in 3D pellet form have higher chondrogenic differentiation capacity in contrast to 3D cell-laden hydrogels through expression of matrix proteins such as SOX9, Col-II and ACAN at 28 days’ time culture. Another group also showed that pellets formed, using both OA and non-OA chondrocytes, have higher matrix protein deposition comparing to chondrocytes cultured on a hydrogel (29). Despite the 3D pellet culture systems provide reliable results, they have limitations namely apoptosis of central cells, poor RNA quality in long-term culture, and only resemble cell-cell interaction neglecting cell-matrix interactions which are highly crucial for clinical applications to restore cartilage defects.

Nevertheless, this approach is suitable to address mechanisms related to chondrocyte differentiation and ECM production under healthy conditions, mainly by focusing on cell-cell interactions (21). 3D micromass systems also successfully mimic hypoxic conditions of native chondrocytes with higher chondrogenic phenotype (31). Mennan et al. demonstrated that hypoxic conditions (2% O_2_, 3D pellets) upregulated SOX9, Col-II, and downregulated activin receptor-like kinase 1 (ALK1), which promoted vascular formation, when compared to normoxia (21% O_2_, monolayer) (32). In other words, Öztürk et al. demonstrated that hypoxic conditions affected 3D-cultured chondrocytes by downregulating the release of β-catenin from the Wnt complex, and, consequently, promoting the chondrogenic phenotype (25). They indicated that chondrocytes cultured under hypoxia expressed higher Col-II, ACAN and GAGs while under expressed Col-I and RhoA which are markers for hypertrophic chondrocytes.

3D micromass models are obtained by using different cell sources, however the first patient-derived tissue grafts were developed based on autologous chondrocytes (33). Tew et al. demonstrated that transduced primary chondrocytes can be expanded to longer passages and successfully re-differentiated into chondrocytes as 3D pellets form (34). However, culture of primary chondrocytes presents issues related to the limited number of cells and high variability obtained from each donor (35). Grogan et al. showed that chondrogenic capacity of 3D pellet models formed using cells from different donors (n=21) can vary depending on the donors through checking the amount of GAG content and expression of CD44, CD151, and CD49c chondrogenic markers as well as the presence of catabolic genes (MMP-2, aggrecanase-2) in *in vitro* conditions (35). They showed that some of the donors expressed less amount of GAG, the above mentioned chondrogenic markers as well as higher number of catabolic genes comparing to other donors. To overcome these variabilities and optimize controlled culture conditions, stem cells, including mesenchymal stem cells (MSCs) mainly derived from bone marrow (BM) placenta, skeletal muscle synovium, synovial fluid and adipose tissue, human embryonic stem cells and recently induced-pluripotent stem cells (iPSCs) have been proposed (36, 37). Each cell type has different advantages and disadvantages, as showed in the **Table 1**. To overcome the disadvantages from monoculture systems, Cooke et al. demonstrated that co-culture of MSCs with chondrocytes could be a promising alternative as chondrocyte hypertrophy is avoided, one of the characteristics of OA that results into cartilage mineralization (40). The chondrogenesis mechanism in co-culture systems is not yet well described, even though it is reported that co-culturing chondrocytes with MSCs led to higher expression of SOX9, aggrecan, and Col-II, and lower levels of MMP-13, Runx2 and collagen-I, when comparing to MSC pellets (40).

**Table 1:** Advantages and disadvantages of different cell types used in joint tissue engineering.

Cell Types	Advantages	Disadvantages
Autologous Chondrocytes (38)	- Low immune-rejection risk	- Donor site variability - Limited number of cells - Uncontrolled chondrocyte dedifferentiation
Mesenchymal stem cells (37, 38)	- Easy to isolate - No limitations on cell number - Higher chondrogenic capacity - Controlled culture conditions	- Potential risk for tumorigenesis. - Low differentiation capacity with increased age - Promotes production of fibrin cartilage instead of hyaline cartilage
Human embryonic stem cells (36)	- Can be differentiated into any cell types derived from ectoderm, mesoderm and ectoderm	- High risk for tumorigenicity and teratoma - Ethical concerns
Induced-pluripotent stem cells (39)	- Easy cell source. - There is no limit in cell number - Promising chondrogenic capacity through reprogramming the synovial cells obtained from OA patients	- Purification of cells into chondrocytes - Genetic modifications: high-cost applications - Teratogenesis perspective - Expensive supplements & regents

OA, osteoarthritis.

#### 3.1.2 Inflamed Joint Model

For better understanding of the joint tissue environment, other joint tissue models, both in healthy and inflammatory conditions, must be highlighted. Kiener et al. established a 3D pellet model by co-culturing synoviocytes and monocytes to mimic synovium using Matrigel^®^ (41). This study was followed by another work established by Broeren et al. They demonstrated that triggering the same micromass model through TNF-α cytokine creates proinflammatory environment and leads to fibrosis and fibroblastic phenotype similar to OA conditions (42). Later, more complex co-culture models have been developed to focus on the crosstalk of different joint tissues under inflammation. Peck et al. successfully established a scaffold-free model to mimic cartilage destruction by co-culturing FLS, macrophages and chondrocytes (43). In this 3D model, chondrocytes were activated through pro-inflammatory macrophages. The expression of ECM proteins (Col-II and Aggrecan) was observed to decrease while ECM degrading enzymes (MMP-1, 3, 13) and inflammatory genes (TNF-α, IL-1β, IL-6) expression increased. Furthermore, this model was also tested for celecoxib which is a highly adopted anti-inflammatory drug for OA patients. Applying celecoxib to the model resulted in decreasing of cartilage damage. Therapeutic effect of the drug was characterized through reduction of MMP-1, MMP-3 as well as increased amount of GAG and collagen. The combination of different cell types has opened new avenues regarding the investigation of the crosstalk between different tissues. However, it should not be neglected that the development of 3D OA models is highly dependent on the physiological relevance and biological accuracy. 3D models mentioned above have focused on cell-cell interactions, without considering cell-matrix interactions until cells produce their own ECM. This issue led to the establishment of scaffold-based 3D models for cartilage tissue engineering.

### 3.2 Scaffold-Based Technologies

#### 3.2.1 Hydrogel-Based

In tissue engineering approach, scaffolds support and induce not only 3D matrix synthesis and organization, but also promote cell adhesion and proliferation (44, 45). Cells may be seeded into the scaffold and then transplanted into the patients to contribute to tissue regeneration at the defected site (46). For this reason, a suitable scaffold should be biocompatible, mechanically stable to allow transplantation, non-immunogenic, permeable to serve as a carrier for growth factors, nutrients and cytokines, allow the promotion of chondrogenic phenotype and, in the end, support cartilage tissue formation (44). In addition to this, it should be biodegradable to assist tissue remodeling by allowing the replacement of damaged tissue with newly generated cartilage ECM (45). Depending on the defect, it might be preferred to be injectable to minimize surgical damage. Scaffold-based therapies have more advantages compared to cell-based therapies. They provide a 3D environment that diminishes chondrocyte dedifferentiation to encourage hyaline cartilage formation. They also provide support on the development of a mechanically more stable tissue that decreases postoperative recovery time (46).

Decellularized matrices and hydrogels are used for joint tissue engineering applications. For instance, cartilage and meniscus derived decellularized matrices have been studied to promote MSCs differentiation into chondrocytes and to stimulate cartilage ECM formation without causing hypertrophic differentiation (47, 48). Visser et al. demonstrated that the presence of decellularized matrices promotes cartilage formation due to biochemical cues, which includes Col-II (49). However, majority of scaffolds for joint tissue engineering applications are hydrogel-based scaffolds. Hence, hydrogels have been incorporated together to provide functional growth of articular cartilage both in *in vitro* and *in vivo* bioreactor systems due to their ability to form irregular shapes and porous structure (50, 51). There has already been certain number of biomaterials that are studied for joint tissue applications. These materials can be classified as natural [collagen, chitosan, alginate, hyaluronic acid (HA), fibrin] and synthetic [Polyethylene glycol (PEG), polylactic acid (PLA), polycaprolactone (PCL), poly(vinyl alcohol) (PVA)] (44). Natural biomaterials have weaker mechanical properties while synthetic materials might trigger toxicity (44). This issue led to the development of composite biomaterials, which aim to offer the best properties from natural and synthetic materials. To form native-like 3D joint constructs using biomaterials, different approaches have been applied, from cell encapsulation *via* simple pipetting to complex 3D bioprinting technologies (52).

Hydrogel-based materials can be combined with cells to obtain 3D systems that may be used as tissue grafts in joint regeneration (53). Encapsulation allows to culture cells in a scaffold that act not only as native-like ECM, but also mechanically supports cells, and promotes cell survival, proliferation, migration and differentiation (54). Hydrogels can be produced *via* two different crosslinking methods: chemical and physical crosslinking. Physical crosslinking occurs due to ionic interactions and hydrogen bonds. Due to the weaker bonds, these interactions are reversible. On the other hand, chemical crosslinking occurs through covalent interactions between polymeric solutions and additives to create irreversible and more stable hydrogels (54). One of the most common chemical crosslinking methods is photo-crosslinking through *Ultraviolet* (UV) and visible light. For instance, the scaffold might be exposed to UV light for a time period to obtain reticulation between the main polymer and photoinitiator (PI) (55). Photoinitiators are substances that produce reactive species, such as cations, anions and free radicals, when exposed to UV and visible light, that allow to obtain photocrosslinked hydrogels. Each PI type has different absorption spectrum wave length, being the most common Lithium phenyl-2,4,6 trimethyl benzoyl phosphinate (LAP), which is activated at 405nm and Irgacure 2959 (IC) is activated at 365nm. Camphorquinone, fluorescein and riboflavin are also widely used and are activated at 450nm, 490nm, and 444nm, respectively (56). These became popular since last decade and are able to finely tune the hydrogel properties in order to obtain the optimal conditions for cells. It has been reported that decreasing PI concentration and UV light intensity results in higher exposure time to form mechanically stable hydrogels (57). Furthermore, the mechanical properties of hydrogels and behavior of encapsulated cells can be controlled through changing certain parameters, such as precursor solution concentration, UV intensity and exposure time (55). Those parameters should be tailored to each cell type to avoid cell toxicity and to provide the necessary microenvironment for, in this case, chondrocyte proliferation, differentiation and chondrogenic phenotype maintenance (55). Otherwise, over crosslinking may result in poor nutrient, growth factor or cytokine diffusion and may lead to the generation of reactive oxygen species (ROS) that induces oxidative stress in cells. On the other hand, a low crosslinking degree may result in lower hydrogel viscosity, and, therefore, poor mechanical stability (58, 59). Several research groups investigated how the material viscosity affected chondrocyte and MSCs behavior through encapsulation of cells in different polymer-based hydrogels (60–64). They indicated that highly viscous hydrogels promoted the retaining of chondrogenic differentiation in both chondrocytes and MSCs, while lower viscosity ones are beneficial for proliferation of chondrocytes. Additionally, Pahoff et al. evaluated the effect of gelatine source [bovine (B) or porcine (P)] and PI type LAP and IC on the hydrogel mechanical properties and cellular behavior of chondrocytes that were encapsulated in GelMA/HAMA hydrogels after 28 days of cell culture (24). They demonstrated that hydrogels formed *via* B-IC displayed higher similarity on compressive strength of native articular cartilage, as well as higher expression of GAGs. Even though the gene expression analysis declared that chondrocytes encapsulated in B-IC showed an increased chondrogenic phenotype, all hydrogel constructs promoted the expression level of Col-II and aggrecan with no difference related to gelatine and PI type. To promote the chondrogenic phenotype additional polymer modifications may be needed. Salinas et al. and Hao et al. encapsulated MSCs in PEGDA hydrogels modified with RGD (Arginine-Glycine-Aspartate) peptide, an integrin binding peptide that is known as promoter of MSCs survival and inducer of chondrogenesis (65, 66). These research groups concluded that RGD peptide modification upregulated the expression of GAGs and Col-II protein *via* blocking the MMP-13 enzyme.

#### 3.2.2 3D Bioprinting

3D bioprinting is a computer-assisted technology that aims to precisely fabricate biological three-dimensional structures in an organized and optimized manner on a layer-by-layer basis, by depositing small droplets or filaments into specific substrates (67). These systems offer to print biological constructs in a desired shape through mixing cells with a solution of natural, synthetic or hybrid biomaterial (mainly hydrogels) and it is called bioink (39). After several iterations along the years, a number of influential researchers in the bioprinting field defined bioink as ‘a formulation of cells suitable for processing by an automated biofabrication technology that may also contain biologically active components and biomaterials’ (68). However, some researchers divide bio-inks into four categories depending on its function: support bioinks, fugitive bioinks, structural bioinks and functional bioinks (69). Support bioinks are the most common bioink type and act as artificial ECM to support cellular behaviour, while fugitive bioinks are temporary materials that can be easily removed by washing or temperature changes. The latter is generally applied when temporary supports are needed or to obtain channels inside the bioprinted structure. To provide mechanical integrity, structural bioinks are often used. These can also have fugitive properties on a long timescale. Finally, functional bioinks are adopted to increase the biomimicry level of the structure by providing biochemical, mechanical or electrical cues. Ideal bioinks should promote healthy cellular behaviour (viability, proliferation, differentiation) to assure functionality of printed tissue, as well as providing necessary mechanical, rheological properties to form a 3D construct (70).

3D bioprinting is divided on the following techniques: inkjet, laser-based, extrusion based bioprinting. Inkjet bioprinting was the first bioprinting technique, and is divided in thermal, electrostatic or piezoelectric inkjet bioprinting (71). Bioinks are ejected based on drop-on-demand technology (DOD) that provides high resolution printing (72, 73). This method offers low-cost production, digital control of highly resolution patterns and high printing speed. However, besides that, it is only suitable for low viscosity and low cell density bioinks (74). This limitation has been solved through laser-based bioprinting, that allows to print high resolution and homogenously distributed cells (39, 75). Laser based bioprinting is a nozzle-free process that works based on the pulsation of laser energy into a cell-containing layer that precisely ejects cell onto a substrate. Although this technique allows to print high-resolution patterns with high cell viability, it is an expensive and time-consuming bioprinting method (73). Extrusion-based bioprinting recently became popular due to the fact that it overcomes some of the limitations of the techniques previously described while allowing to print fiber sized constructs offering possibility to mix different bioinks (76). This technique works based on the deposition of bioink through a nozzle driven by pneumatic pressure, or a piston (72). However, this technique also has some disadvantages such as low resolution, deformation of construct and poor cell viability due to the increased shear stress that occurs during the extrusion process (77). Despite those advantages and disadvantages of all bioprinting techniques, they have been accepted as reliable systems in terms of cell viability and proliferation (67).

3D bioprinting technologies have been used for joint tissue applications and hydrogels are commonly used as bioinks for 3D bioprinting approaches due to their rheological behaviour, biocompatibility, and mechanical properties (78). Collagen, HA, gelatine, chitosan and alginate are the most adopted polymers on bioinks for printing cartilage tissue (79). Bioink viscosity must be suitable for each bioprinting technique to ensure printability and maintenance of the shape of the post-printing construct (80). Hence, the previously discussed parameters for hydrogel crosslinking are also critical for bioprinting technologies. This way, Markstedt et al. developed a nano fibril cellulose (NFC) and alginate based bioink to obtain optimal viscosity for chondrocyte culture (79). They concluded that their bioink (NFC/Alginate; 80:20) showed stable mechanical properties, when comparing to both before and after crosslinking, and further promoted cell viability after 7 days of culture. However, this study did not include any task regarding chondrogenic phenotypic characterization. Singh et al. performed a study on the development of silk-gelatine based bioink for cartilage tissue engineering applications (78). They successfully developed a crosslinker-free silk-gelatine based bioink with the required rheological and mechanical properties for printing. This material also promoted biocompatibility on both *in vitro* and *in vivo* after 14 days. Their bioink boosted the chondrogenic phenotype (ECM proteins: Col-II, SOX9, Aggrecan, GAGs) while decreasing cell’s hypertrophy. Lam et al. studied GelMA and hyaluronic acid methacrylate (HAMA) based hydrogel scaffolds regarding cell viability, morphology and chondrogenic phenotype for two different cell densities through stereolithography based bioprinting (81). They found that high cell density cell-laden GelMA hydrogels expressed more ECM proteins (Col-II and Aggrecan) at the end of 14 days of culture. It was also demonstrated that addition of HA to GelMA increased the polymer viscosity, provided more controlled, and homogenous bioprinting, and promoted expression of GAGs and Safranin (82). Duchi et al. introduced an apparatus called ‘Biopen’ that allowed to print viable adipose stem cells inside a hydrogel shell formed by GelMA and HAMA. This device is expected to be a promising tool for printing of 3D structures directly into chondral defects to promote *in situ* cartilaginous tissue formation (57, 83). **Table 2** summarizes the most common materials for different joint tissue models.

**Table 2:** Examples of currently available scaffold-based *in vitro* models for cartilage tissue applications.

Material		Joint tissue model	Cell type	Crosslinking method	Fabrication technique	Major observations/findings	Drawbacks	Refs
Name	Properties							
Gelatine: GelMA	Viscous, stiff Young’s modulus: 30.3~ kPa Gel-to-sol phase:<37°C	Cartilage	Chondrocytes	Photocrosslinking	Simple pipetting	Higher viscosity promotes the retaining of chondrogenic phenotype of chondrocytes, while low viscosity encourages cell proliferation	No data presented regarding localization of ECM proteins	(61)
Gelatine: GelMA	Young’s modulus: from 3.8~ to 29.9~ kPa Swelling ratio of hydrogels is inversely proportional with stiffness	Cartilage	Chondrocytes	Photocrosslinking	Simple pipetting	The influence of hydrogel stiffness was investigated by altering methacrylate ratio of gelatine Higher stiffness encourages expression of GAG, Col-II, Safranin as well as rounder morphology of cells after 14 days of culture	Longer culture time is needed	(62)
PEGDA/GelMA	Stiffness: from 1.6 kPa to 25 kPa Hydrophilic and bio-inert	Cartilage	Mesenchymal stem cells	Photocrosslinking	Simple pipetting	Differentiation of MSCs into chondrocytes was improved with higher stiffness hydrogels	No data presented regarding localization of ECM proteins	(63)
GelMA/HAMA	Viscosity >300 Pa and it inversely proportional with applied shear stress Gel-to-sol phase: 22°C	Cartilage	Adipose stem cells	Photocrosslinking	Extrusion-based bioprinting	Provides handheld printing of scaffolds with/without cells on the wound site during surgery	Not possible to print less than 1mm sized defects Need for temperature stability due to user’s hand	(57, 83)
GelMA & HAMA	Viscous, 5% GelMA-1% HAMA mix	Cartilage	Chondrocytes	Photocrosslinking	Stereolithographic bioprinting	Higher cell density encapsulated in GelMA hydrogels showed more chondrogenic phenotype First study that utilizes HAMA in a stereolithographic bioprinting approach to produce cartilage-like tissue *in vitro*.	Properties of HA such as solution viscosity that varies by temperature affected cell viability and hydrogel itself showed autofluorescence during imaging process.	(81)
Cellulose/Alginate (C/A)	Water content: 97.50% (w/v) Gel-to-sol phase: 25°C Viscosity varies depend on the C/A ratio	Cartilage	Chondrocytes	Chemical crosslinking	Droplet based bioprinting (micro valve)	High cell viability (~86%) of chondrocytes after 7 days of culture; Mechanically stable & biocompatible bioink establishment	Does not include any assay that explored the chondrogenic phenotype of cells	(79)
Silk−Gelatine	Gel-to-sol phase: 31°C Storage modulus: 1.5 kPa Degradation rate:60% in 28days	Cartilage	Chondrocytes	Crosslinker-free	Extrusion-based bioprinting	Biocompatible bioink for both *in vitro* and *in vivo* Suitable rheological & mechanical properties before & after printing	Longer culture time is needed	(78)
GelMA/HAMA	Compressive modulus is divergent with gelatine, PI type; higher using bovine sourced gelatine and Irgacure	Cartilage	Chondrocytes	Photocrosslinking	Simple pipetting	Different gelatine sources and PI types were investigated. B-IC showed more chondrogenic phenotype through RT-PCR, but no significant difference between ECM proteins through immunofluorescence staining	More evidence is needed to conclude their findings	(24)
GelMA & HAMA	Compressive modulus: GelMA:26kPa; HAMA:96kPa;	Cartilage	Chondrocytes	Photocrosslinking	Simple pipetting into a Teflon mold covered with glass	Different polymers were evaluated regarding mechanical properties and chondrogenic differentiation. GelMA hydrogels found to be mechanically more stable and to highly promote the expression of GAGs, Col-II, Aggrecan while downregulating MMP-13, PRG4 and Col-I after 21 days of culture	Actin filament staining revealed that morphology of chondrocytes was not round shaped as described in literature	(84)
GelMA modified with cartilage-derived matrix (CDM)	GelMA modified with CDM has higher compressive modulus comparing to only GelMA (range between ~120-175kPa)	Osteochondral	Chondrocytes & Multipotent stromal cells	Photocrosslinking	Simple pipetting	CDM particles were found to stimulate the formation of a cartilage template. GelMA was almost fully degraded after the subcutaneous implantation of engineered osteochondral construct	Cell viability assay and experimental set up were tested with different cell densities	(49)
Alginate	No information regarding mechanical properties of scaffold	Cartilage	Chondrocytes	Chemical crosslinking	Micropipetting using syringe pump	Highly organized 3D alginate scaffolds were produced by droplet-based microfluidic device. Cartilage-like structures were formed after 4 weeks implantation.	Expression of chondrogenic ECM markers started to decrease after 4 weeks of culture, therefore more animal study is needed	(85)
Fibrin, functionalized with TGF-β3	More viscous comparing to the control group	Cartilage	Stromal cells	Chemical crosslinking	Simple pipetting	*In vivo* tests revealed that the developed injectable fibrin hydrogel promoted chondrogenesis, when comparing to control group (gelatine microspheres)	Further studies are needed to understand how particle size of ECM proteins affects chondrogenesis.	(86)
Collagen	Biocompatible, physiologically relevant microenvironment	Cartilage	Osteoarthritic chondrocytes	Chemical crosslinking	Water-in-oil emulsion method	Osteoarthritic chondrocytes encapsulated in the 3D collagen microsphere better recapitulated the OA phenotypes comparing to the pellet culture.	The *in vitro* model should be further exposed to inflammatory cytokines and/or mechanical loading for better understanding of OA responses	(87)
PEG	Biocompatible, physiologically relevant microenvironment	Cartilage	Chondrocytes and Macrophages	Photocrosslinking	Simple pipetting	Two cell types were encapsulated separately but cultured in the same medium in a Transwell system to understand the crosstalk between cartilage and macrophages in OA. Chondrocytes in co-culture with activated macrophages expressed higher MMP-1,3,13 and IL-1β, TNF-α, comparing to monocultured chondrocytes and chondrocytes co-cultured with non-activated macrophages.	No data presented regarding localization of ECM proteins	(88)

GelMA, Gelatine-methacryloylate; kPa, kilo Pascal; ECM, extracellular matrix; °C, Celsius; GAG, Glycosaminoglycan; Col-II, Collagen-II; PEGDA, Poly(ethylene glycol) diacrylate; MSCs, mesenchymal stem cells; Pa, Pascal; HAMA, hyaluronic acid methacrylate; HÁ, hyaluronic acid; C/A, Cellulose/Alginate; w/weight/volume; PI, photo initiator; B-IC, bovine Irgacure; RT-PCR, real time polymerase chain reaction; MMP-13, matrix metalloproteinase; PRG4, Proteoglycan 4; CDM, cartilage-derived matrix; 3D, 3-dimensional; TGF-β3, transforming growth factor beta-3; IL-1β, Interleukin-1 beta; TNF-α, tumor necrosis factor-alpha.

Besides scaffold-based 3D *in vitro* models, tissue explants have also been investigated as they provide cells with their natural environment (3). Grenier et al. established an *in vitro* OA model through collagenase treatment of cartilage explants and by applying mechanical stress on the tissue (89). They demonstrated that collagenase treatment provided degradation of cartilage ECM in the superficial and intermediate zones of cartilage tissue, which was confirmed through histological analysis. To create more complex models, a cartilage–synovium explant co-culture model was developed by Beekhuizen et al. (90). Their results indicated that cartilage tissue co-cultured with synovium tissue from the same donor decreased GAGs expression when comparing to monoculture. Additionally, this group observed a similar pro-inflammatory cytokine expression in both conditions. Even though tissue explant models provide cells with similar physiological environments, availability of donor and/or intra-donor variability between samples limits the application of tissue explants in the field. These models are able to provide a proper environment similar to the *in vivo* situation, however they cannot incorporate the physiological stimulations of cartilage tissues, such as continuous nutrient supply and, more importantly, mechanical stimulations. In that view, more advanced cell culture platforms have been investigated, namely bioreactors and organ-on-chip systems.

#### 3.2.3 Bioreactors

Bioreactors are devices that enable to culture cells in a controlled environment (e.g., temperature, pH, nutrient supply, mechanical stimuli). Spinner flasks, rotating wall vessels, and perfusion bioreactors are examples of bioreactors that have been designed based on tissue and application. To mimic shear, compression, and hydrostatic pressure of healthy joint, Park et al. developed a bioreactor that applied compression and shear stress 1 hour per day on an engineered cartilage construct which was produced by encapsulating chondrocytes in a fibrin/hyaluronic acid hydrogel (91). This research group concluded that their engineered construct expressed more GAGs and Col-II which were horizontally parallel to the stimuli side when comparing to static group. To understand the interplay between bone and cartilage, Schwab et al. established an osteochondral culture platform that comprised two separated compartments to allow the seeding of bone and cartilage derived cells in one platform (92). This platform was able to provide tissue-specific growth factors to each cell type during long-term cultures (over 84 days) while maintaining the cartilage matrix content and its mechanical properties. Despite the fact that this osteochondral model provided new insights regarding the interaction between tissues, it is still limited to investigate the interplay between bone and cartilage for longer culture time.

## 4 Advanced 3D Models: Microfluidic Technologies

### 4.1 Organ-on-a-Chip

**Figure 2:**
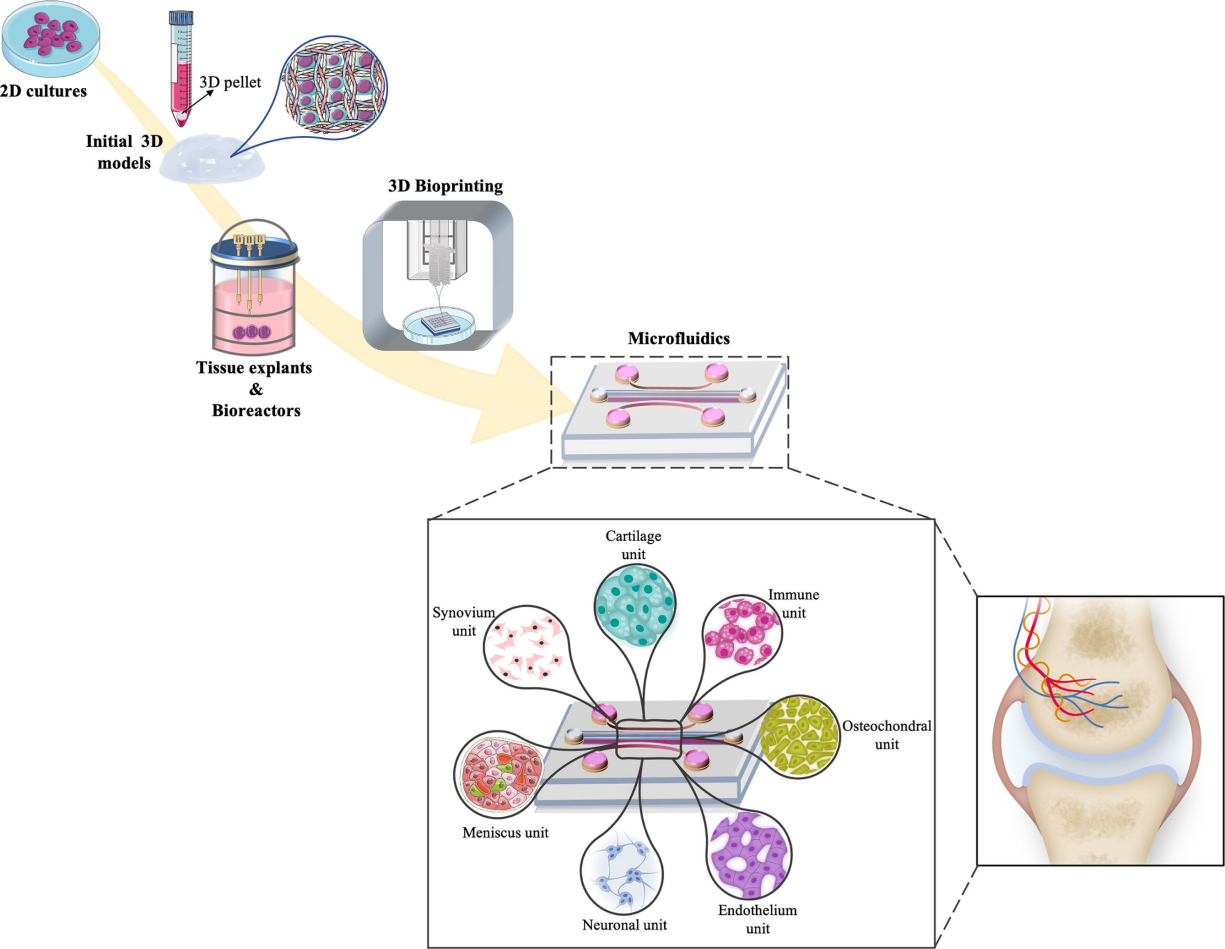
Schematic representation of the timeline evolution of different *in vitro* models for cartilage tissue engineering strategies. Increasing complexity can be observed starting from 2D simple culture, 3D pellets models, bioreactors and bioprinting applications into more multifaceted platforms as microfluidic models that comprised of joint-on-chip components (synovium, cartilage, immune, osteochondral, endothelium, neuronal and meniscus unit) and how these components match with the native joint environment.

Microfluidic systems are described as suitable tools to study several tissues in one device by being able to recapitulate both healthy and pathological conditions (93). Microfluidic systems allow to use low amount and highly controllable reagents, as well as integrating sample separation and detection such as cell culture, sorting and/or cell lysis (94). One of the most common microfluidic systems in tissue engineering applications is OoC platforms (95). Schematic of currently available *in vitro* models, starting from 2D simple culture until microfluidic technologies and possible components of joint-specific OoC platforms are shown in **Figure 2**. Advantages and disadvantages of above-mentioned *in vitro* models are described in **Table 3**.

**Table 3:** Advantages and disadvantages of currently available *in vitro* models.

Current *in vitro* models	Advantages	Disadvantages
2D Monolayer culture (96)	- High reproducibility - Simple experimental set up	- Results with dedifferentiation of chondrocytes - Simple experimental set up
3D Cell-based (96)	- Higher chondrogenic capacity than 2D models - Successful recapitulation of cell-cell interactions	- Ignores cell-matrix interactions - Enable to mimic only one tissue type - Variabilities depending on the donors
3D Scaffold-based (96)	- Offers a native like microenvironment - Enable to mimic only one tissue type	- Low mechanical properties - Difficult to investigate the interplay between multiple tissue components
Tissue Explants (3)	- Cells are maintained in their native microenvironment - Provides to investigate physiology of whole tissue	- Limited number of donors. - High donor variability
Bioreactors (3)	- Enable cultures cells in a controlled environment - Longer culture time	- Do not provide fully dynamic platforms
Microfluidics (3)	- Physiologically relevant *in vitro* model - Cost-effective due to low volume of reagent	- May result with uncontrolled fluidic flow due to bubbles in the channels - Limited experimental analysis due to low amount of sample

2D, 2-dimensional; 3D, 3-dimensional.

Organ-on-chip systems incorporate cell culture setups with up-to-date techniques to create more reliable 3D microenvironments mimicking physiological, chemical and mechanical conditions of native tissues. These platforms pave the way by mimicking and regulating key tissue parameters, such as concentration gradients, shear force, cell patterning, tissue-boundaries and tissue–organ interactions, in one device (94). They are one of the most promising alternatives to animal models for mimicking the biological, mechanical and chemical environment of native tissue creating dynamic culture conditions (97). These devices may be fabricated by soft lithography technique or 3D printing technologies, being polydimethylsiloxane (PDMS) one of the most common used polymer to replicate these devices (98). They are comprised of different size and shape micro features that allow controlled delivery of oxygen and nutrients to cells to overcome hypoxia and nutritional deficiency of 3D cellular structures for promoting cell adhesion and proliferation (99). These systems allow to use, not only lower cell numbers, but also very small working volumes of biological and chemical reagents. On the other hand, to ensure the necessary cellular microenvironment to promote cellular behaviour to recapitulate 3D environment of joint tissue in *in vitro* conditions, hydrogels can be used inside the OoC platforms.

OoC platforms have been evolving rapidly in the past decade, coupling previously defined technologies (iPSCs, biomaterials, bioreactors, 3D bioprinting) to model both healthy and diseased conditions related to multiple organs and tissues (100). There are several organs replicated on-chip over the past decade, including lung, liver, heart, intestine, muscle, vessels, bone etc. These have been developed for applications such as toxicology assessment, vascularization and drug testing on tissue-specific functions of those tissues (100). These developed models allow to study basic mechanism of represented organ physiology and disease. The ultimate aim of the OoC platforms is to develop human body-on-chip combining individually developed organ models in a single device will allow to investigate the crosstalk between different tissues/organs in human body.

Many of recently established OoC models includes multiple compartments to study interactions of different cell types of represented organ/tissue model. The critical point of connecting different compartments is optimization of culture conditions for each cell types such as culture media or fluid flow along channels (101). This multicompartment design is highly suitable for developing *in vitro* joint models, where multiple cells sourced from different joint tissues can be cultured in one device and can be exposed to different mechanical and/or chemical stimuli through fluidic flow inside the channels. For example, cells presence in synovial joint are naturally exposed to shear forces by flow of the synovial fluid, so these cells will perform better under such stimuli (102). Additionally, fluidic flow along the channels also brings possibility to recapitulate OA-inflammatory environment through the circulation of proinflammatory cytokines or immune cells (96).

### 4.2 Joint-on-Chip

Musculoskeletal disorders are driven by unbalanced crosstalk between various tissues, such as muscle, joint and nerves, including different cellular and molecular pathways related to the tissues (93). To understand the biophysical and biochemical mechanisms behind those diseases, animal models have been widely used. However, animal experimentation is not cost effective, presents variability between samples, and brings ethical issues that slow down the development of clinical trials (103). To this regard, joint-on-chip systems could provide a more predictive, cheaper, ethical, and faster approach that is driving the development of organ on a chip and organ on a plate technology. These systems incorporate several advantages at different levels: i) biological: use of human cells, ‘personalizing’ the chip; relevant long-term culture conditions; study the crosstalk between different cells; creation of dynamic environments using perfusion to create physical and chemical gradients (95); ii) setup: easy to use; reproducible conditions; controlled microenvironment through microsensors integration; continuous monitoring and live data recording; and iii) economical: low cost fabrication; scalable; high throughput; need for very small reagent volumes (103). The establishment of physiological and pathological cartilage models comprising microfluidic technology are here described.

**Figure 3:**
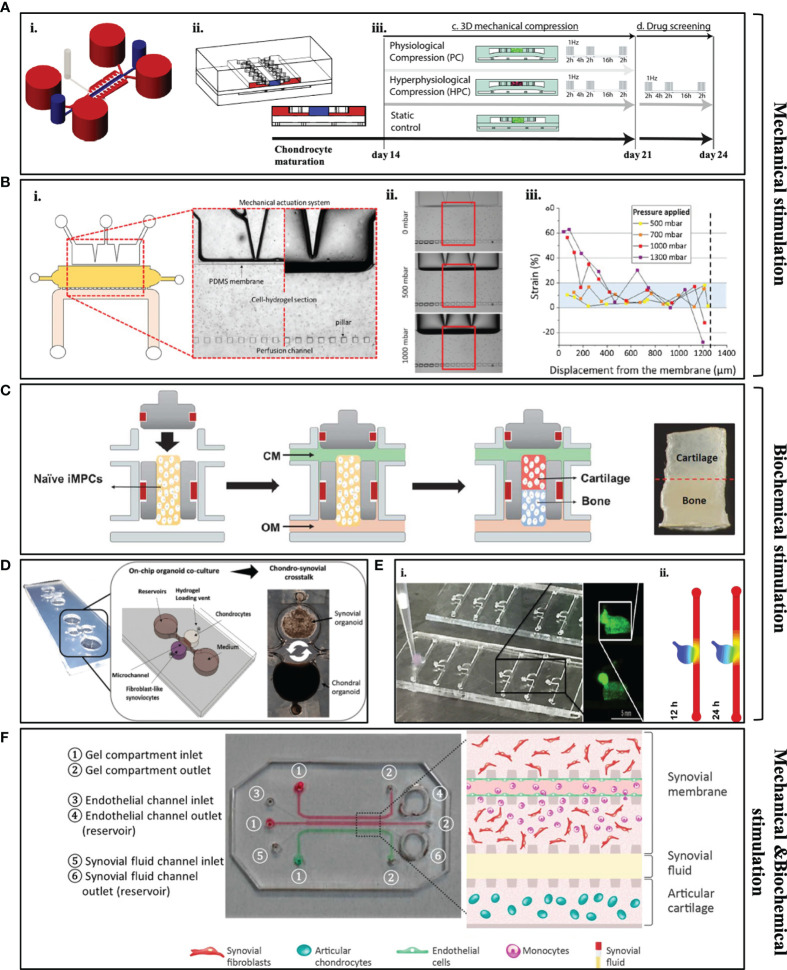
Examples of some of the currently available microfluidic models. **(A, B)** Mechanically stimulated microfluidic models. **(A)** i: schematic representation of the microfluidic platform to mimic strain compression; ii: lateral cross section of the device; iii: movement of PDMS membrane under different mechanical compressions. [Adapted from (104)] **(B)** i: Design of the microfluidic platform includes PDMS membrane to create mechanical pressure; ii: light microscopy images of how PDMS membrane moves under different pressures; iii: quantitative results of displacement level of the membrane related to applied pressure [Adapted from (106)]. **(C–E)** Biochemically stimulated microfluidic models. **(C)** Design of the osteochondral microfluidic model that includes encapsulated IPSCs in order to form bone and cartilage tissue [Adapted from (107)]. **(D)** Design of the organoid-based joint-on-a-chip co-culture system that includes chondral and synovial organoids [Adapted from (108)]. **(E)** i: Real picture of the cartilage-on-chip model and overview of the encapsulated chondrocytes in a hydrogel scaffold; ii: stimulation for the diffusion of biomolecules through circular chamber and channel (Adapted from (109). **(F)** Components of the synovium-on-chip model and schematic representation of the developed joint microenvironment [Adapted from (110)].

OoC platforms allow to recapitulate native interstitial fluidic environment and simulate shear stress of the joint in *in vitro* conditions to understand cartilage physiology, both in inflammatory and healthy conditions. The approach to the full joint-on-chip models include the already described different structural parts of the joint in separately devices, such as synovium-on-chip, menisci-on-chip, OA-on-chip or combining more than one of those systems in one platform. OA-on-chip include two major characteristics that set them apart from the other cartilage 3D models: mechanical loading and biochemical stimuli through immune mediators to engineer cartilage tissue (104). It has been defined that mechanical systems (load-control or actuation systems) have the potential to the mimic biomechanical environment of OA without excessive inflammatory cytokines (105). Mechanical loading systems are also able to influence the expression of ECM proteins and lubricin to levels similar to the ones found in healthy hyaline cartilage tissue (91). Occhetta et al. investigated the effect of hyper physiological compression on chondrogenic phenotype (104). This group established a microfluidic platform to mimic controlled strain compression similar to OA patients by using a mechanical actuation system to understand the pathology of the disease (**Figure 3A**). In this work, the healthy cartilage microtissue was firstly achieved by encapsulating primary human articular chondrocytes in PEG hydrogel for 14 days and characterized through expression of matrix proteins (Col-I, II and ACAN) and quantification of glycosaminoglycans (GAGs). Later, mechanical compression was applied to the mature cartilage microtissue through PDMS membrane for 7 days and this mechanical compression resulted in downregulation of Col-I, II and ACAN, and upregulation of catabolic enzyme (MMP-13) and proinflammatory cytokine (IL-8) protein expressions as it is expected in OA phenotypes. Here, the model was not only characterized by the expression of catabolic enzymes and inflammatory markers, but also by the analysis of the chondrocyte hypertrophy. For example, the developed OA *in vitro* model showed upregulated Col-X expression and IHH expression and downregulation of GREM1, FRZB and DKK1 genes that act as antagonists for BMP and Wnt signaling pathways that correlates with OA characteristics. This model was addressed a drug-screening tool after 14 days of chondrocytes maturation, by exposing the established OA-model with different anti-inflammatory/anticatabolic compounds (IL-1 receptor antagonist, Rapamycin and Celecoxib). It was observed that all of the compounds significantly reduced both MMP-13 and IL-8 expression which were correlated with the drug concentration, when comparing to healthy cartilage model. Additionally, Paggi et al. investigated the effect of mechanical actuation on OA through the use of a PDMS membrane inside microfluidic platform to create mechanical stimulation (106). Cartilage tissue construct was developed by encapsulating primary human chondrocytes from OA patients inside agarose hydrogels (**Figure 3B**). Here, different mechanical compression parameters were daily applied daily to the construct through PDMS membrane to mimic healthy (800mbar) to hyper-physiological (1000mbar) conditions of cartilage tissue. Even though this platform was successfully able to mimic the shear strain that is found in articular cartilage using a positive pressure controller, longer culture time is needed to evaluate the protein and matrix deposition of chondrocytes.

In contrast to above mentioned mechanical actuation systems, Lin et al. successfully established an OA model through immune mediators to investigate the crosstalk between bone and cartilage in a dual-flow bioreactor (**Figure 3C**) (107). Induced pluripotent stem cells (iPSCs) were differentiated into chondrogenic and osteogenic lineages and cultured in two-different compartment of the chip, separated by GelMA hydrogel. After 28 days, iPSC-derived osteochondral (OC) microtissue was exposed to an inflammatory cytokine [interleukin-1b(IL-1β)] to create an inflammatory environment similar to OA. Under inflammation, it was revealed that bone is critical to promote cartilage hypertrophy in the joint. After creating an OA-like environment in the cartilage compartment, inflamed cartilage was treated with Celecoxib, a non-steroidal anti-inflammatory drug and a COX-2 inhibitor which is commonly targeted during OA treatment. Celecoxib treated OC microtissue demonstrated lower expression of catabolic and proinflammatory cytokines (MMP-1,2,3,9,13; IL-6, IL-1β and ADAMTS4) in the cartilage compartment as well as upregulation of ECM proteins (Col-II and ACAN) through real-time polymerase chain reaction (RT-PCR). Thus, this model serves as a potential high-throughput drug screening platform for OA models. Rosser et al. also developed the “microfluidic nutrient gradient-based model”, a healthy cartilage-on-chip model, which was developed by encapsulating primary equine chondrocytes in fibrin hydrogel. Initially, a healthy cartilage microtissue model was validated checking expression of matrix proteins (Col-II, SOX9 and ACAN) through quantitative PCR and the healthy microtissue model was exposed to inflammatory cytokines (TNF-α and IL-1β) for 24 hours to recapitulate the chondrogenic phenotype of OA patients in a cartilage on-chip model (**Figure 3E**) (109). Inflammatory phenotype and hypertrophic differentiation of chondrocytes were confirmed through upregulation of MMP-1,3,13, aggrecanase and Col-X. The biochemically injured *in vitro* model was later exposed to steroid treatment. As expected, this treatment resulted with upregulation of the ACAN expression while downregulation ECM degrading enzymes.

For better understanding of the joint pathology, multi-tissue-based devices, which include cartilage, synovium, subchondral bone and/or menisci, have been developed. Lin et al. developed a 3D osteochondral microfluidic model with two tissues (111). The H-shaped design of the device allowed to form chondral tissue on one part of the device and bone tissue on the other side of the platform with differentiating BMSCs inside GelMA/HAMA hydrogels to those specific cell types. After 4 weeks of culture, they successfully characterized both compartments of the device and both tissue compartment were separately treated to pro-inflammatory cytokine (IL-1β) for 7 days. It was observed that, after cytokine treatment, ECM degrading enzymes (MMP-1, 3, 13) were upregulated in both compartments of the device when comparing to an untreated engineered construct (accepted as healthy model). This also resulted in downregulation of ECM proteins expression (Sox9, Col-II, and Aggrecan) on cartilage compartment. However, no protein localization was specified through immunochemistry. In another study, the role of macrophages and neutrophils in an osteoarthritic knee have been investigated by combining both mechanical and immune regulators (110). Here, Mondadori and colleagues developed an articular joint microfluidic *in vitro* model that was comprised of two compartments (cartilage and synovium) separated by a channel for the synovial fluid (**Figure 3F**). The joint-on-chip model was established culturing of primary OA-patient derived articular chondrocytes and synovial fibroblasts in fibrin hydrogel. To mimic the cartilage environment, shear stress has been applied along the microchannel walls to create diffusion of chemokines from the synovial fluid to the synovium compartment. This microfluidic platform is of high importance since it provides information regarding the extravasation of monocytes into synovium and is able to identify the activated macrophages and neutrophils on the engineered OA construct.

To investigate the arthritic joint microenvironment, Ma et al. developed a microfluidic platform that allowed to mimic FLS migration and invasion-mediated bone erosion through co-culturing commercially available human synovial sarcoma cells and mouse BMSCs which were differentiated into osteoclasts previously (112). This study revealed that in the co-cultured group, Cadherin-11 and RANKL were overexpressed by the synovium cells and osteoclasts, respectively. This model was also tested with an anti-inflammatory drug, called Celastrol which prevents FLS activation and diminishes bone erosion. It was demonstrated that FLS migration is reduced as well as expression of Cadherin-11 and TRAP activity are decreased after 4 days of co-culture compared to untreated control groups. Thus, this model may be used an effective anti-RA drug screen model for targeting FLS migration-mediated bone erosion. In arthritic joint, abnormal angiogenesis occurs in the subchondral area and macrophages and FLSs are thought to be the first triggers of inflammation-induced angiogenesis (113). Additionally, the level of angiogenesis is correlated with the degree of the inflammatory potential of FLSs in the joint (114). To this regard, another study was established by Lozito et al. to understand the pathogenesis of OA. Here, a 3D osteochondral microtissue that was surrounded by the endothelium to mimic vascularization in OA was developed (115). This model included bone, osteochondral interface, cartilage and synovium respectively from bottom to top, organizing in different layers. The bone compartment was peripherally enclosed by the endothelium component in a collagen-I hydrogel scaffold. This work revealed that crosstalk between the endothelium and bone cells promote the release of fibroblast growth factors, IL-1, and IL-6. These systems allow to move forward on studies regarding intravasation of surrounding tissues and to understand how blood vessels affect the behaviour of fibroblast-like synoviocytes and macrophages in the arthritic joint. A recently published study by Rothbauer et al. aimed to understand the crosstalk between synovium and cartilage tissue of arthritic joint (108). Here, for the first time, a patient-derived dual organoid model was successfully established by co-culturing primary human synovial fibroblast obtained from RA patients and commercial human primary chondrocytes (**Figure 3D**). Co-culturing of chondral organoids with synovial organoids resulted in higher arthritic diseases characteristics, such as increased expression of IL-6, IL-8, VGEF and MMP-13. This microfluidic device allows to overcome several limitations by fully mimicking of arthritic joint microenvironment as well as offering drug testing studies and personalized medicine applications. A detailed classification of currently available joint-on-chip models is presented in **Table 4**.

**Table 4:** Currently developed joint-on-chip models.

Joint model	Cell types	Stimulation type	3D ECM material	Drug Testing	Major observations	Refs	Drawback
Rheumatoid Arthritis	FLSs & BMSCs	Biochemical stimulation	No	Celastrol	Allows to mimic FLS migration and invasion-mediated bone erosion in RA. Drug testing inhibited migration and erosion markers.	(112)	No data presented regarding the pain caused by Musculoskeletal disorders
Rheumatoid Arthritis	FLSs & chondrocytes	Biochemical stimulation	Matrigel for FLSs Fibrin for chondrocytes	No	First established chip-based chondro-synovial dual organoid model	(108)	
Cartilage	Chondrocytes	Mechanical stimulation	Fibrin/Hyaluronic acid	No	The engineered construct has similar Young modulus and capability to express GAG, Safranin and Col-II proteins to native cartilage tissue	(91)	
Osteoarthritis	Chondrocytes	Mechanical stimulation	Agarose	No	The device was separated through PDMS membrane that allows to create mechanical stimulation on chondrocytes. Chondrocytes cultured closer to hyperphysiological stimulation side have less cell viability. More data is needed on chondrogenic behaviour under mechanical stimulation	(106)	
Osteoarthritis	Chondrocytes	Mechanical stimulation	PEG	Il1Ra & R2apamycin	Successful recapitulation of mechanical stimuli on OA patients Suitable platform for drug testing studies.	(104)	
Osteochondral	IPSCs	Biochemical stimulation	GelMA	Celecoxib	Bone is critical to promote cartilage hypertrophy in the joint. Therapeutic effect of the drug was confirmed in OA conditions	(107)	
Osteoarthritis	Chondrocytes	Biochemical stimulation	Fibrin	Steroid treatment (triamcinolone)	Healthy joint model characterized through expression of SOX9, Col-II, Aggrecan Inflammatory conditions were diminished after the steroid treatment	(109)	
Cartilage & Synovium	FLSs & chondrocyte & HUVECs	Biochemical & Mechanical stimulation	Fibrin	No	Successful extravasation of monocytes and identification of activated macrophages and neutrophils from the engineered OA construct	(116)	

3D, 3-dimensional; ECM, extracellular matrix; FLS, fibroblast-like synoviocytes; BMSCs, bone marrow stem cells; RA, Rheumatoid Arthritis; GAG, Glycosaminoglycan; Col-II, Collagen-II; PDMS, Polydimethylsiloxane; PEG, Poly-(ethylene glycol); Il1Ra, Interleukin 1 rapamycin; IPSCs, Induced pluripotent stem cells; GelMA, Gelatine-methacryloylate; SOX9, SRY-Box Transcription Factor 9; HUVECs, Human Umbilical Vein Endothelial Cells; OA, Osteoarthritis.

Even though it is not as common as OA and RA, it is indicated that Infectious Arthritis (IA) may also results in musculoskeletal pain if it is not fully treated (117). IA occurs when the infection from any part of the body spreads to the joint or synovial fluid that surrounds joint that also affecting the nervous system. In many cases, IA is caused by bacteria that usually infect human through insect bites [i.e. Staphylococcus aureus and Borrelia burgdorferi sensu lato that cause Lyme’s disease (117)] as well as some viruses (Parvovirus, Alphaviruses, Hepatitis B &C, Epstein-Barr virus and Zika virus) (118). Currently, patients who undergo IA are recommended to use antibiotics, however there are no FDA approved treatments that ensure full recovery after late stage of the disease (117). In this regard, researchers have been focusing on the development of early diagnosis techniques using microfluidic technologies. Nayak et al. developed a rapid lab-on-a-chip point of care (PoC) assay for the serologic diagnosis of human Lyme disease (119). The assay named as mChip-Ld test and includes ELISA and western blot techniques to identify antibodies against the Borrelia burgdorferi bacterium in 15 minutes. Nayak and colleagues detected three antigens namely rP100, PepVF, rOspC-K that can detect antibodies specific to the bacteria with the sensitivity between 35–56% for Early Stage I, 73–77% for Early Stage II and 96–100% for Late Stage III of Lyme’s disease. Another research groups have been using microfluidic technologies for early diagnosis of Staphylococcus aureus that also cause IA (120, 121). Song et al. developed a microfluidic platform that works with fluorescence labeling principle of the bacteria that helps to detect antigen-antibody interaction easily. Kalashnikov et al. developed a rapid microfluidic based research model that can be a potential *in vitro* screening model to study different types of bacteria and their antibiotics (120). Here, the group aimed to create enzymatic stress in bacteria due to the presence of β-lactam antibiotic. The antibiotic caused damage on the bacterial cell wall, which resulted in cellular death that was detected by using the fluorescence dye Sytox Green.

## 5 Conclusion and Future Perspectives

Up to this date, researchers have developed different *in vitro* models to understand the interplay between different joint tissues and to move forward on the development of novel therapeutic treatments for patients. Recent advances in biology and engineering enable to develop and recapitulate 3D dynamic microenvironments to produce more reliable *in vitro* joint models. 3D microfluidic systems are positioned in the forefront of the joint tissue engineering field by allowing the integration of mechanical, biochemical, and physical cues in one platform. Besides all progresses that have been achieved, mimicking the whole joint tissue in one device remains highly challenging.

Currently developed platforms are mostly focused on mimicking the microenvironment of OA through mechanical stimulations or inflammatory mediators without considering neural players which leads to pain in OA patients (16, 106, 109). To better understand the mechanism behind the OA-associated pain, neuroimmune expression profile must be addressed. Compartmentalized microfluidic platforms allow to investigate the interactions between neuronal cells and non-neuronal cell types, including bone cells, muscle cells, chondrocytes, etc. (122). For example, coculture of sensory neurons with osteoblasts (123, 124) and dental pulp stem cells (125) has been successfully established using two-compartment microfluidic devices to understand the innervation of particular peripheral tissues. Additionally, different groups have used two-compartment microfluidic platforms as a tool to understand the innervation profile of skeletal muscle cells (126, 127). To date, these studies showed that *in vitro* culture systems are expected to be a versatile tool for elucidating molecular players that lead to peripheral innervation. Hence, it is possible to address different questions regarding signaling pathways using compartmentalized microfluidic devices under specific physiological and pathological conditions of OA (122). The use of microfluidic *in vitro* models could also offer the conditions to recapitulate circulatory immune cells environment under dynamic flow. These *in vitro* tools are expected to eliminate the complexity of living organs/tissues studies, still integrating enough variables of different systems and providing accurate experimental conditions. Only by developing versatile and accurate *in vitro* tools we will be able to unravel the mechanisms behind OA disorders and provide innovative therapeutic approaches.

## Author Contributions

EK, RR, ML, and EN contributed to the selection and organization of the topics to include in the document. EK wrote the first draft of the manuscript and elaborated the tables and images. EK, RR, and EN wrote sections of the manuscript. All authors contributed to manuscript revision, read, and approved the submitted version.

## Funding

This project has received funding from the European Union’s Horizon 2020 research and innovation program under grant agreement No.860462.

## Conflict of Interest

The authors declare that the research was conducted in the absence of any commercial or financial relationships that could be construed as a potential conflict of interest.

## Publisher’s Note

All claims expressed in this article are solely those of the authors and do not necessarily represent those of their affiliated organizations, or those of the publisher, the editors and the reviewers. Any product that may be evaluated in this article, or claim that may be made by its manufacturer, is not guaranteed or endorsed by the publisher.
